# Social Media as Online Shelter: Psychological Relief in COVID-19 Pandemic Diaries

**DOI:** 10.3389/fpsyg.2022.882264

**Published:** 2022-05-30

**Authors:** Ran Feng, Yulei Feng, Alex Ivanov

**Affiliations:** School of Media and Communication, Shanghai Jiao Tong University, Shanghai, China

**Keywords:** social media, pandemic diary, individual narrative, psychological relief, COVID-19 pandemic

## Abstract

The pandemic diary on social media is a special form of online communication. Studying individual narratives in social networks during the pandemic and post-pandemic periods can help us generate valuable knowledge about the behaviors of media users and the function of social media in a public health crisis. This research focuses on psychological relief in virtual public spaces and explores how social media individual narratives affect people’s psychological health in a state of emergency from the perspective of narrative theory. Based on 19 in-depth interviews with Chinese diary writers, it has been found that the narrative genres of the pandemic diary were mainly Restitution and Quest narrative, while a few were categorized as “Restrained chaos” narrative. The purpose of editing pandemic diaries is to communicate both inwardly and outwardly. The pandemic diary can promote self-relief, public communication, emotional drive, meaning connection, and identity construction in public spaces, thus helping shape a sense of unity and belonging, and facilitating the psychological reconstruction of people who are vulnerable to potential mental health crises.

## Introduction

The COVID-19 pandemic has had a tremendous impact throughout the world since the end of 2019, and one which has been felt in every corner of the internet. Living in the pandemic period, people are responding in various ways, including expressing emotions and creativity (Belli and Alonso, 2021). Millions of users in every country have left a plethora of comments on social media with a variety of intentions and sentiments.

Disasters are often interpreted as a cause of immense physical and psychological pain to a minority but have consequences for the majority as well (Huang, 2016). Although the felt emotions are often private, the way these emotions can be expressed depends on society’s power structure. When traditional media reigned supreme, state-oriented narratives suppressed individual communication in the past. However, today’s social media have empowered nearly every single member of society to voice their opinion. What used to be hidden now bubbles up to the surface. Social media and digital platforms facilitate information-sharing, user-created content, and collaboration among people (McFarland and Ployhart, 2015). The upward spiral of collective emotional activation has attracted widespread attention (Gerbaudo, 2016).

The narratives in the pandemic provide us with a novel perspective to understand public health practices (Zhao, 2021). By observing the journaling behavior on social media, this research analyzes the content in these posts and the embedded identity interactions through narrative theory. It attempts to discover and summarize the rules of interaction between different subjects (diary writers and readers) in the process of emotional exchange.

## Literature Review

### Media Narrative Theory

Mass media is a product of collective social participation (Anderson and Curtin, 2002). When it comes to user-generated content in social media, individuals not only present their knowledge of past experiences but also focus on self-cognition and reconciliation (Shao, 2014). During the COVID-19 pandemic, it was widely prevalent for social media users to share their experiences as they related to this predicament. As a representative of historical narrative text, the pandemic diary has the characteristics of originality and authenticity that fictional text does not possess (Aldrich and Eccleston, 2000; Jackson, 2005; Skultans, 2007).

As an effective way to convey symbolic meaning, narrative profoundly affects the process of public understanding and interpretation of meaning (Caldiero, 2004). The individual narrative is a prose in which a witness describes and comments on an event on the basis of their individual presence (Chen, 2020). Scholars attach great importance to the role of social media in studying crisis narrative and the narrative of disease (Moulton, 2016). Social media platforms encourage everyone to participate in the production of individualized narratives, and the public becomes the main narrator in crisis.

This study draws on the theory of narratology as particularly relates to pain and disaster narratives. American sociologist Frank (1995) proposed three types of disease narratives in the western context: Restitution, Chaos, and Quest narrative (see Table 1). This classification enables us to study how personal, social, and cultural factors affect individual narratives, as well as help patients’ friends and family and health service providers and researchers to better understand the experience of illness (France et al., 2013). Many researchers have applied the theory to the narrative of physiological diseases, such as breast cancer (Nettleton et al., 2004; Thomas-MacLean, 2004; Pinnock et al., 2011), but paid little attention to the narrative of potential psychological disorders. Yet people’s state of mind could easily collapse when a serious public health crisis suddenly occurs, leading to major and long-term mental illness. It is necessary to classify and explore narratives related to mental trauma during the COVID pandemic time based on Frank’s narrative framework. Moreover, this study tries to find new narrative categories outside of this framework and supplement existing literature. Hence, the first research question could be raised:

**Table 1:** Frank’s narrative genres.

Genres	Defining characteristics
Restitution	Illness is temporary, not a permanent threat to mortality, a transitory interruption, able to construct oneself as good as “new” or recovered, I am or will be fine, expect cure or remedy
Chaos	Despair, depression, futility, hopelessness, vulnerability, impotence, powerlessness, lack of control, no one in control, uselessness, no recognition for pain and suffering, emotional battering, lack of temporal order (unless told retrospectively)
Quest	Seek alternative ways of being ill; accept illness; emphasize gains from illness experience; see illness as an opportunity, opening, or challenge; sense of purpose; includes branch genres of memoir, manifesto, and auto-mythology
Quest memoir	Simply accept illness, incorporate illness into life, trials told stoically, no special insight gained from illness experience
Quest manifesto	Demands for social reform or social action, special insight gained from illness experience
Quest auto-mythology	Change of character, personality, rebirth, self-reinvention

*RQ1*: What are the narrative genres of pandemic diaries in Chinese social media in the context of the COVID-19 pandemic?

### Psychological Pain and Relief During the COVID-19 Pandemic

The COVID-19 pandemic has disrupted the normal order of society and caused tremendous psychological pressure. Had no intervention measures been taken, the psychological shock would have resulted in cataclysmic proportions (Bao et al., 2020). Post-disaster mental disorder has always been a research focus of psychological health communication in China and abroad. Some scholars believe a disaster event is always beyond the ability of ordinary people to cope with, thus causing a series of mental health problems, such as post-traumatic stress disorder, major depression, and even suicide (Zhao et al., 2009). When faced with disasters, individuals experience both personal and collective trauma. Sudden personal trauma attacks an individual’s psychological defense mechanisms, while collective trauma wreaks havoc on the relationship between sufferers and others (Zhang and Wang, 2008). Mental disorders caused by pandemics also tend to exist for a prolonged time. Although victims can return to the normality of their pre-trauma everyday life, many suffer from avoidance syndrome and emotional numbness especially in the face of new stressful events in the future (Dyke et al., 1985). Medical professionals often compare COVID-19 with SARS given their similar genetic sequence and dissemination pattern. The dissemination of SARS in 2003 caused widespread panic, anxiety, and other psychological problems in Chinese society (Xie et al., 2005). Individual responses vary depending on multiple individual personality traits, such as self-efficacy and social support (Fan, 2003).

White and Epston (2013) proposed Narrative Therapy, according to which the practice of writing is a key mechanism to help patients rediscover the meaning of life. One positive aspect of today’s digital environment is that social media allow people to express themselves with various forms of recording. For example, Knight et al. (2015) stated that during the treatment of chronic and non-communicable diseases, social media has the potential to help design healthy behaviors and improve treatment effects, and enable patients to interact with peers in a safe and confidential manner. Social media and narrative therapy have also helped African-American adolescents withdraw from drug addiction (Qureshi et al., 2015). Most of these studies, however, explored the psychological relief of social media in Western contexts, and there is a dearth of such research on Chinese media. The current study fills this gap by addressing how individuals seek emotional comfort on social media:

*RQ2*: What is the purpose of generating individual diaries on social media during the pandemic?

Research on disease narrative has discovered the role of multiple parties in the process of sharing (Ziebland, 2004). As it initiates new forms of network and interactivity, social media is effectively reshaping our cognition of disease (Gonzalez-Polledo and Tarr, 2016). Paton and Irons (2016) found that since the information needs of victims of natural disasters are often left unsatisfied, they rely on social media to obtain more information. The narrative of the disaster would also help to build faith and stimulate civilians to actively participate in post-disaster construction. This study focuses on the emotional release of users on Chinese social media during the COVID-19 pandemic and tries to evaluate the potential of psychological healing from individual narratives. Hence, here comes our third research question:

*RQ3*: What changes have been brought about by the individual narrative writing on social media in the context of the pandemic?

## Research Method

### Data Collection

The “pandemic diary” term used in this study refers to content about daily life events, personal thoughts, and emotions social media users post in relatively frequent intervals on the internet during the pandemic. They usually appear in single or mixed types of text, pictures, and short videos on social media, such as Sina Weibo. This research mainly collects text-based diaries, as well as a small number of pictures and video diaries. The number of words in each text diary varied from eight to 2,973 words, and the length of the video ranges from 59 s to 12 min and a half. The whole collection of diaries contains 103 pictures and 131 videos.

### In-depth Interviews

The respondents of this study were Chinese social media users who kept on posting pandemic diaries on WeChat, Sina Weibo, Bilibili, etc. Nearly half of them came from Wuhan, where the pandemic initially broke out. Respondents’ identities were diverse, and their diaries were relatively continuous and complete. In order to ensure differentiation, researchers mainly looked for typical cases and selected convenient samples as a complement to make a comparison. The researcher sought the help of office staff from the Wuhan Municipal Government to contact respondents. In the meantime, researchers sent invitations on social media to recruit participants. Research participants have been asked to complete a study-specific consent form. The information of respondents is shown in Table 2.

**Table 2:** Basic information of participants.

No.	Name	Age	Gender	Current occupation	Current residence	Marital status
1.	JC	28	Female	Nurse	Shijiazhuang, Hebei, China	Unmarried
2.	ZZW	25	Female	Nurse	Shijiazhuang, Hebei, China	Unmarried
3.	XXW	67	Male	Retired	Wuhan, Hubei, China	Married
4.	LK	26	Female	Real estate consultant	Wuhan, Hubei, China	Unmarried
5.	DYQ	25	Female	Teacher	Wuhan, Hubei, China	Unmarried
6.	XM	21	Female	Student	Wuhan, Hubei, China	Unmarried
7.	ZYC	31	Male	Financial analyst	Wuhan, Hubei, China	Married
8.	DXJ	27	Female	Student	Seoul, Korea	Unmarried
9.	MY	45	Male	Administration staff	Wuhan, Hubei China	Married
10.	XJ	43	Female	Teacher	Wuhan, Hubei, China	Married
11.	ALX	33	Male	Accountant	Sydney, Australia	Married
12.	WJ	21	Male	Student	Xiamen, Fujian, China	Unmarried
13.	CM	41	Male	Photographer	Yangzhou, Jiangsu, China	Married
14.	DCR	20	Female	Student	Wuhan, Hubei, China	Unmarried
15.	JMS	45	Male	Freelancer	Sydney, Australia	Married
16.	XW	20	Female	Student	Yangzhou, Jiangsu, China	Unmarried
17.	MSL	26	Male	Product manager	Xianyou, Fujian, China	Unmarried
18.	DHH	24	Female	Student	Yantai, Shandong, China	Unmarried
19.	XDR	33	Male	Freelancer	Taiyuan, Shanxi, China	Unmarried

The research conducted semi-structured in-depth interviews *via* WeChat with 19 participants who persisted in writing pandemic diaries on social media. These respondents were mainly medical staff, patients’ relatives, online and offline volunteers, and college school students. The diaries of some respondents have been reposted by influential online media, and are quite representative. The discussion guide consisted of 10–20 questions, as is detailed in the supplementary materials for interview questions.

### Ethnographic Content Analysis

Diary extracts were documented and interview audio files were transcribed, checked for accuracy, and anonymized by the research team. Two researchers inductively identified codes in the data according to the theoretical background of narrative genres and constantly compared the diary extracts and interview transcripts to identify sub-themes.

We have completed an ethnographic content analysis, which is a qualitative-oriented method to code respondents’ diaries and interview data. According to Altheide (1987), ethnographic content analysis is designed to examine or supplement prior theoretical works by obtaining categorical and unique data in order to develop analytical constructs appropriate for investigations. This study has referred to previous research (Vickovic et al., 2013; Belli and Alonso, 2021) to make structured qualitative content analysis and triangular validation of the coding schemes.

**Figure 1:**
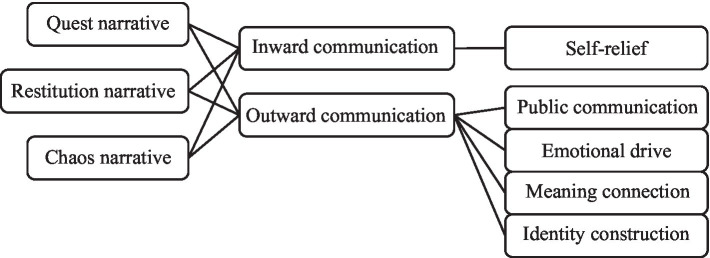
Theoretical frame construction.

Data were subsequently coded through open and axial coding methods to explore linkages between the data and systematically indexed into an initial coding scheme. These coding techniques are widely used in qualitative research for developing grounded theory (Strauss and Corbin, 2014). We applied the open coding method to divide documents into smaller sections or paragraphs, mark similarities and differences and give codes to similar contents or categorize them into a higher abstract class. Then, axial coding was conducted to integrate the relationship between concepts and categories developed from the open coding process. Researchers imported the data into the qualitative analysis software NVivo11 to find connections and establish theme s. Tables 3 and 4 elaborate on specific aspects of the coding process. And next, we visualized the theoretical frame construction on the basis of the coding result. Figure 1 presents major themes and marks associations emerging in the coding procedure.

**Table 3:** The axial coding scheme of pandemic diary narrative.

Main category	Sub-category	Supplementary explanation
Quest	Quest memoir	Quest memoir is the basis of Quest narrative
	Quest manifesto	Quest manifesto is the advanced stage of the Quest narrative
	Quest auto-mythology	Quest auto-mythology is the ultimate goal of Quest narrative
Restitution	Positive psychological construction	Positive psychological construction is the premise of the Restitution narrative
Chaos	Normal chaos	Express negative emotions unreservedly
	Restrained chaos	Express negative emotions with reservation

**Table 4:** The open coding scheme of in-depth interview content.

Example	Conceptualization	Categorization
I want to say something to myself online about what I usually do not want to say offline.	Satisfying self-talking needs	Inward communication
Although photos do not match texts, they can help me recall many things.	Recording precious memories	
I tried my best to tell myself that it was not my fault for things to become like this, and I tried to ease my negative emotions.	Releasing psychological pressure	
I post information about the country where I am studying in, such as reminding people not to travel to Korea shortly.	Posting anti-pandemic progress	Outward communication
My family members are worried about my physical and mental condition. To reassure them, I post positive and relaxed content on WeChat.	Informing personal condition	
The internet also has a bright side. For example, the number of infected people can be updated every day, and the knowledge of prevention can bring hope to people.	Providing positive information	
To show to others what we have done because volunteers do a lot of work.	Hoping to get attention	
I only write what I see, and it is enough to comfort myself.	Comforting oneself	Self-relief
I told my relatives and friends not to spread rumors and cancel the Spring Festival gathering.	Reminding relatives and friends	Public communication
I share stories about helping stray cats on social media.	Sharing warm feeling	Emotional drive
SOS messages are very close to me, and I can feel their hardship.	Seeking help for empathy	Meaning connection
The fight against the pandemic made me feel concerned for us from all walks of life and made me believe that my career choice is correct.	Identifying with one’s mission	Identity construction

## Findings

### Narrative Genres

The pandemic diaries written by most respondents were not confined to a single genre, and each had one or two dominant narrative genres. A majority of respondents mainly used the Restitution and Quest genre to give positive hints of self-healing. Only four respondents posted content resembling the Chaos genre.

The Quest narrative was indeed the most common, as 17 respondents applied it in their diaries, while auto-mythology rarely appeared. The process begins with mere acceptance of the disaster, transitions to a determination to take action, and ends with personality changes, such as undertaking some meaningful action as a diversion from the pandemic. For example, ZYC (aged 31) wrote that he decided to start a new translation job after being a volunteering driver:

*“I found a new job last night. A large number of medical resources arrived in Wuhan. Volunteering translators were in need. I’m good at professional English in biochemistry, and my wife has passed the Japanese N1 test. Our translation skills are good enough. No hesitation!”* (ZYC, aged 31)

The main function of the Restitution narrative is to build self-confidence and encourage others. Though suffering from physical and psychological pain, positive storytellers firmly believe that society will prevail (Belli and Alonso, 2021). The diaries of five respondents were optimistic as they thought that all the difficulties would soon be overcome. As JC (aged 28), a nurse, wrote: “As long as everyone has a strong faith, we will definitely win”; while XXW (aged 67), retired, noted: “I believe that Wuhan people will surely defeat the pandemic! I want to show my will and confidence to everyone.”

CM (aged 41), a photographer, and DCR (aged 20), a college student both explicitly mentioned in their video strong beliefs in their country, the Communist Party of China, and the government. While afraid of the pandemic, they did not believe humans would be defeated by the virus. These users also expressed hope to transmit confidence and determination through their diaries. This sense of hope caused an emotional contagion that protects viewers from personal collapse (Belli and Alonso, 2021).

None of the analyzed diaries had typical characteristics of a Chaos narrative. This genre often fails to express coherent meaning, as authors often lack control over their emotions which are often imbued with negativity. Participants in this study clearly expressed their thoughts and emotions. Negative sentiments were sometimes present but always restrained. We deemed it appropriate to name this genre “Restrained chaos” as evidenced by the following quote.

*“It’s useless to make a phone call for help. Even if you call the emergency number, you can’t wait for more than 400 numbers, and there is no car to take the patient to the hospital. One day in the early morning, my friend’s family tried many ways to ask for a single bed in the hospital, but they always got stuck. I didn’t know what to say, and I didn’t know what else I could do, except to say sorry to her. I seemed to be a waste. That was the first time I felt the pandemic was so close to me.”* (LK, aged 26)

This Restrained chaos narrative reflects LK’s feeling of powerlessness in a messy situation. It was posted on her Weibo account, which had more than 200 followers. It seems likely she tried to conceal her irritation toward the administrative inefficiency as well as self-blame for her inability to cope. In a follow-up interview, this participant admitted she had better be more careful in controlling her negative emotion due to the “openness” of the Weibo platform.

The Restrained chaos narratives were uncommon in video diaries; the latter were generally less negative than text. As XW (aged 20), a student, noted, “When recording videos, I cannot help expressing a positive attitude, but I am afraid that I would be too optimistic, which will cause no one to pay attention to the severity of the pandemic.” The confusion faced by this vlogger demonstrates that the presence of bystanders inhibits the expression of emotions.

### The Purpose of Writing: Inward and Outward Communication

The purpose of writing generally falls into two categories. First is “one-way inward communication,” a kind of author’s self-talk, regardless of whether the diary is read or watched by others. Many participants felt a strong desire to communicate with themselves during the pandemic. The diaries posted on social media are permanent records, and they serve to remind authors of an unforgettable tragedy (XM, aged 21; ZYC, aged 31; XXW, aged 67). Keeping a diary also enables pandemic victims to voice their pain and anxiety and attempt to alleviate their depression. The expression of these emotions varied among participants. Some directly poured out their annoyance or anger (LK, aged 26; DYQ, aged 25); others used sarcastic and self-deprecating rhetoric (MY, aged 45); and one participant said she used the diaries to simply sort out her thoughts and be more rational (LK, aged 26). Yet another said he replayed a video in order to “figure out the psychology trait at the time and estimate the tolerance level” (XDR, aged 33). For authors who applied the Restrained chaos narrative, the public nature of social media prompts them to notice whether their words and deeds are appropriate and whether their statement is objective, rather than go to an extreme.

The second category of purpose is “two-way outward communication” as evident in Quest manifesto, Quest auto-mythology, and Restitution narratives. The individual narrative in the epidemic constructs the interactive relationship between the narrator and the audience, which further strengthens the transformative role of the individual narrative (Chen, 2020). More than half of the respondents assumed that their diary would be read or watched by others. Supposing that friends and family could be part of this audience, the authors tended to focus on reassurance (JC, aged 28; ZZW, aged 25; XJ, aged 43). Respondents who assumed the diary were followed by strangers expressed the intent of acting as spiritual models by spreading positive beliefs online (ZYC, aged 31; JC, aged 28; ZZW, aged 25). All respondents held the belief that the public nature of diary writing on social media outweighed privacy concerns. Some even saw the pandemic experience as a harbinger of a single collective memory where individual privacy is a thing of the past. The majority of posters believed in sharing the struggle with conquering the virus.

### The Result of Writing: Psychological Relief in Social Media

#### Self-Relief

As in other media, diaries in social media can also promote emotional expression and self-encouragement. Whether subtle encouragement or an unrestrained outpouring of emotion, the individual narrative is an effective way of psychological reconstruction. Respondent LK (aged 26) had come to the following realization:

*“Most stay-at-home people made a limited effort during the pandemic. As long as I can express my willingness to help, that is enough. Keeping a diary allows me to understand what I am actually able to do and forces me to calm down.”* (LK, aged 26)

The self-reflective process of these diaries often leads to the realization of how limited people are in their response to the pandemic. Respondent MY (aged 45) saw his role as one to prevent residents from going outside frequently. He used a sarcastic expression “I was speechless” referring to the frustration he experienced at his office between the different styles of conflict resolution. Because life during the lockdown period was tedious, respondents JMS (aged 45) and WJ (aged 21) both regarded filming video diaries as an indispensable part of their schedule. Apart from expressing one’s emotions, the continuous production of the pandemic diary could enhance people’s self-discipline and stimulate introspection. As another respondent mentioned, “The pandemic has made me reconsider what life means to us, why we work so hard, and why we are always anxious” (ALX, aged 33). The unforgettable life experience of the pandemic makes them truly value what is important and necessary and what is not. For these participants, the diary is a coping mechanism that helps them co-exist with negative emotions. A kind of cognitive-emotional redefinition of the situation develops and prepares them to face illness and death emotionally (Belli and Alonso, 2021).

#### Public Communication

In the background of national self-quarantine, social media has offered an opportunity for the public to communicate with family and friends online. Public narratives on social media are often more accessible and readable than professional medical narratives. Individual narratives strike a balance between fact and feeling, and foster awareness and participation (Li, 2020). For instance, two nurse respondents had urged friends and family not to fabricate and disseminate rumors and encouraged them to cancel their gatherings around the Chinese Spring Festival. These two nurses shouldered the responsibility of communication between the medical department and the general public. When offline communication is unavailable, as is often the case with such disasters, diary writing on social media provides a chance to share joy and pain together. Respondent DHH (aged 24) mentioned she was always touched by the friendly comments that were posted below her video, especially many people saying “Welcome home.” Another respondent, MSL (aged 26), admitted that his quarantine experience was significantly improved by the comments he regularly read on the screen. Respondent ZYC (aged 31) regarded a diary as a way to boycott abuse and rumors on the internet. In the early days of the epidemic, there were many malicious messages slandering Wuhan people on social media, so he wanted to use his personal experience to tell the audience that people in Wuhan were actively saving themselves, and they were not the so-called “walking virus.” In just a few days, he received thousands of likes and hundreds of encouraging messages and comments.

#### Emotional Drive

During the pandemic, a community of shared destiny has been formed among individuals, families, and society. The pandemic diaries of all participants have attracted varying degrees of attention and praise in social circles. They described the various situations during the pandemic by recording their personal lives. Pandemic diaries are often presented as situational narratives, and a strong emotional connection is established between the diary’s author and readers by creating specific situations. DYQ (aged 25), a volunteer working in a small community, often posted stories about helping stray cats and dogs with her colleagues on WeChat. The description of trivial detail displayed a simple but warm emotional experience, which moved a lot of readers. The presentation of the pandemic diary on social media not only contains textual narratives, but also achieves vivid and intuitive audio-visual effects by uploading pictures and videos, creating a real sense of presence for readers. Among all the diaries, whether selfies of medical workers wearing masks, showing volunteers delivering vegetables to the community on a daily basis, or scenery outside the window of a stay-at-home person, all of them represent a positive and persistent individual effort in the pandemic. They have caused deep emotional vibration and brought faith and hope to others. Emotions spread online can be transferred through emotional contagion, which leads people to experience the same emotions, or to form emotional patterns, without awareness (Belli and Alonso, 2021).

#### Meaning Connection

Pandemic diaries often include concrete descriptions of changes happening to individuals who share similar experiences. Two nurse respondents recorded their daily work in their diaries. The story fragments demonstrated the significance of national medical assistance. ZZW (aged 25), a nurse providing aid in Wuhan, found the reading of her diary not only relieved her anxiety and fear about the pandemic, but also made her firmly believe in her career choice. Photographer CM (aged 41) imagined himself as an amateur reporter and recorded the real world in the eyes of ordinary Yangzhou citizens who were not quarantined, hoping to convey a different voice, “in order to allow people who are not in this city to understand more about the city’s situation.” These contents have made the audience of the diary more deeply aware that the pandemic is a reality that everyone is facing. This sense of commonality connects everyone and renders the construction of unity.

#### Identity Construction

Many respondents stated that they have received unexpected attention and support in journaling. DYQ (aged 25), a volunteer who served in a community, mentioned that voluntary work was difficult to carry out in the early stage and often had to face the unreasonable demands and abuse by rude civilians, which made her desperate to solicit the understanding of friends on the online platform. After she posted the diary, many people comforted her through thumbs-up and comments, which brought her great support. In the interview, she said:

*“The comments are very reassuring, and they try to evaluate the whole thing objectively, by telling me that what I have done was right. I feel that there is still someone by my side, and I will feel better.”* (DYQ, aged 25)

In the narrative of the fight against the pandemic, people are often trapped in thinking about the value conflicts between either self and others, or individualism and collectivism. Therefore, it is necessary to gain recognition of self-worth through communication with others. This also helps to soothe the psychological pain they have suffered during the pandemic, so as to let them find the meaning of life and create a sense of belonging.

## Discussion and Conclusion

Catastrophic events can be viewed as opportunities for social transformation, bridging social inequalities, and strengthening individual competencies (Baker, 2009). Since every person could be regarded as an expert in terms of dealing with his or her biographical pandemic situation, it was suggested to shift the focus from the specialists to everyday life “professionals” in order to fully understand the crisis (Boldt, 2021). Journaling in virtual media space is a distinctive form of social media communication. Research on pandemic diaries helps us better understand social media users’ behavior and examine social media’s function. This research focused on psychological relief in public spaces, specifically analyzing how individual narratives would affect people’s psychological status in a health crisis.

Firstly, this article explored the genres of individual narratives in the context of the COVID-19 pandemic. According to the qualitative content analysis of participants’ diaries, the narrative genres are mainly Restitution and Quest narrative, among which Quest memoir and manifesto are more common than others. In the diaries collected in this study, no one completely matched the Chaos genre, but we suggest the existence of a “Restrained chaos” genre. This narrative reveals negative emotions, but the authors acknowledge the openness of social media platforms thus posting less harsh content. Frank (1995) found that patients would use different types of narratives at different phases of the disease. Similar to that study, we found that at different stages of the pandemic, the diary authors presented different narrative types. Meanwhile, the interviewees may also apply more than one narrative genre at the same phase. Therefore, the narrative of the pandemic diary is more complicated than we expected. In this research, fewer participants presented Chaos narratives in their diaries, as chaotic narratives are difficult to capture. The most negative narrative genre in public space usually hides in trivial moments of life, as noted by Frank (1995). Regardless of whether the author wanted their diary to be read by others, they would strive toward proper expression. In addition, only a small number of respondents used the Quest auto-mythology narrative. The possible reason is that this genre involves fundamental changes in individual identity and personality. People need to make a comprehensive self-reform after gaining deep insights into the pandemic, which requires a lot of effort. The narrative in individual diaries basically helps participants recognize and discover themselves in the backdrop of the pandemic, and stimulates resilience which is understood as an innate trait of people and systems (Houston and Buzzanell, 2018).

Next, this study discussed the purpose of writing pandemic diaries on social media. We summarized the purposes as inward and outward communication based on in-depth interviews. Not all participants assumed they had an audience. Authors without such expectations just focused on internal dialog, whereas those who presumed an audience expected more interpersonal interactions. All participants tend to believe that social media diaries are more public than private. The findings are in line with a study that revealed marked resilience and a willingness to benefit others as a result of the lockdown (van de Groep et al., 2020). The results demonstrate the need for discovery of oneself and to develop altruistic behavior through inward and outward communication.

Finally, the study focused on the results of diary editing on social media during the pandemic. It was found that pandemic diaries could promote self-relief, public communication, emotional drive, meaning connection, and identity construction in public spaces. Inward communication desire is satisfied by self-relief generated in journaling, while outward communication encourages the other four positive changes. These results may indicate that people’s psychological resilience may be higher than it was anticipated by numerous mental health professionals, at least in the non-clinical populations (Moroń and Biolik-Moroń, 2021). During pandemic times, people may focus more on family and friend relationships, or on creating a sense of solidarity with other people, which may help to alleviate anxiety and enhance confidence, commitment, and social bonding. Previous research found the emotional impacts appeared more pronounced in the narratives of female participants (Scott et al., 2021), our study also echoes this. The mentality of most diary authors changed positively after journaling, as the diaries helped shape a sense of unity and emotional belonging.

Social media is a platform that emphasizes interactivity, often with emotional content. They connect the users’ emotions and influence them (Saldias and Picard, 2019). This appears to be certainly the case with the pandemic diary. Exploring the underlying reasons behind it may supplement research on emotional communication in social media. From in-depth interviews with participants, we found most of them believe online diaries are different from private ones. Online posters may hold back their dissatisfaction and anger for personal impression management reasons and for altruistic concern not to flare up panic. Due to the presence of the imagined audience, the communication of emotion in social media also has the characteristics of domestication, which hides the noise that is not conducive to stability and unity and amplifies the harmonious voice (Huang, 2016). Some scholars have stated that emotional expression has social rules, which means the power and class structure of society determine the expression and suppression of emotion (Hochschild, 1979; Kemper, 1980). This research also supports this claim to a certain extent. This conclusion may have some limitations because most participants write diaries on social media with acquaintances. If they are in a completely unfamiliar network environment, perhaps the diary can break through the pressure of face and authority, and convey a voice with more public awareness and objective thoughts.

In the process of transformation into a platform society, diversification and centralization have become important features of network platforms, that is, multilateral relations can be carried out through organizations (Hu, 2018). Examining the public’s ritual construction of disasters on social media, we found that the arousal and drive of emotion is a micro but important component. The praise of the heroes, the gratitude to the volunteers, and the tenacious confidence in defeating the pandemic trigger a strong sense of cohesion and solidarity in Chinese society. In the symbolic space constructed by social media, people share certain text symbols to express real emotions, and collective consciousness also emerges (Fan, 2011; Huang, 2016). Therefore, we believe that the public space in the age of social media is conducive to shaping a sense of unity and emotional belonging, and constitutes a space of digital togetherness (Marino, 2015). Commitment to solidarity is invoked when people acknowledge the pandemic as a collective threat and are told by authorities that “standing together” is always effective to mitigate the hazard (Guttman and Lev, 2021). As a special narrative mode, the pandemic diary places personal experiences in a wider scope and conveys individualized experiences to the public. Just as Buzzanell (2010) put forward the process of building resilience after disasters, the healing effect of pandemic diaries is inseparable from the collaborative communication between different groups. Psychological relief is always indispensable from the participation of members of families, workplaces, communities, and organizations. The narrative of the pandemic diary constructs collective memories and emotions in this special period of time (Garro and Mattingly, 2000). The stories in the diary are continuously reproduced, linking people of different classes and interests to encourage a series of social actions (Zhou, 2020), so that individuals suffering from disasters and traumas no longer feel isolated and helpless.

Social media platforms have made pandemic narratives an immediate product. They turn painful and optimistic fighting experiences to be real and sensible and arouse much empathy. Interactivity has established a supportive community through countless personalized expressions. We believe that identifying the ways that pandemic diaries can build resilience related to the psychological burden is important, but we are also aware of a more critical reading of this. Our life experiences are perspectival and can deeply distort reality, truth, and objectivity (Goldie, 2012). The online environment allows a greater sense of freedom in expressing oneself and less concern related to judgment (Saladino et al., 2020). The narratives of the pandemic on the internet are not always true, because diary writers try to weaken negative expression for the sake of impression management (Goffman, 1959), or deliberately spread discrimination, resentment, and false information to attract attention. Therefore, the psychological relief in the pandemic diary is somewhat limited, and it may also cause certain harm to the author. For instance, the hero narrative tends to be potentially problematic for making healthcare workers feel ashamed and embarrassed (Halberg et al., 2021). In order to make individual narratives in social media play a more active and positive role in the context of health crises, we need to create a good online environment and attach more importance to supervision issues, only in this way can diary editors express themselves legally and reasonably.

This study is not without limitations. We collected and analyzed the diaries and interview manuscripts mainly in the Chinese context. Since the COVID-19 pandemic will always be a global issue, it would be better to include more research materials from diverse cultural backgrounds. Due to the unpredictable changes in the pandemic situation, psychological consequences can be long-lasting (Ho et al., 2020). It is necessary to study further the long-term psychological relief in pandemic diary narrative. We suggest that future quantitative studies focus on this perspective, it is important to help future generations of psychologists and patients to collaborate on the potential benefits of keeping the journaling behavior on social media, as a kind of education and training on the benefits and effectiveness of telepsychology (Maheu et al., 2012). Hopefully, the habit of writing pandemic diaries online would be an effective supplementary intervention of online psychological services in severe public health emergencies.

## Data Availability Statement

The original contributions presented in the study are included in the article/supplementary material, further inquiries can be directed to the corresponding author.

## Ethics Statement

The studies involving human participants were reviewed and approved by the Institutional Review Board of Shanghai Jiao Tong University. Written informed consent to participate in this study was provided by the interview participants. Written informed consent was obtained from the individual(s) for the publication of any potentially identifiable images or data included in this article.

## Author Contributions

RF and YF: study design, data collection, data analysis, and paper writing. AI: study design and manuscript review. All authors contributed to the article and approved the submitted version.

## Conflict of Interest

The authors declare that the research was conducted in the absence of any commercial or financial relationships that could be construed as a potential conflict of interest.

## Publisher’s Note

All claims expressed in this article are solely those of the authors and do not necessarily represent those of their affiliated organizations, or those of the publisher, the editors and the reviewers. Any product that may be evaluated in this article, or claim that may be made by its manufacturer, is not guaranteed or endorsed by the publisher.
